# Giant Malignant Phyllodes Tumour of Breast

**DOI:** 10.1155/2014/956856

**Published:** 2014-12-04

**Authors:** Ramakrishnan Krishnamoorthy, Thejas Savasere, Vinod Kumar Prabhuswamy, Rajashekhara Babu, Sadashivaiah Shivaswamy

**Affiliations:** Department of General Surgery, Victoria Hospital, Bangalore Medical College and Research Institute, Bangalore, India

## Abstract

The term phyllodes tumour includes lesions ranging from completely benign tumours to malignant sarcomas. Clinically phyllodes tumours are smooth, rounded, and usually painless multinodular lesions indistinguishable from fibroadenomas. Percentage of phyllodes tumour classified as malignant ranges from 23% to 50%. We report a case of second largest phyllodes tumour in a 35-year-old lady who presented with swelling of right breast since 6 months, initially small in size, that progressed gradually to present size. Examination revealed mass in the right breast measuring 36*×*32 cms with lobulated firm surface and weighing 10 kgs. Fine needle aspiration cytology was reported as borderline phyllodes; however core biopsy examination showed biphasic neoplasm with malignant stromal component. Simple mastectomy was done and specimen was sent for histopathological examination which confirmed the core biopsy report. Postoperatively the patient received chemotherapy and radiotherapy. The patient is on follow-up for a year and has not shown any evidence of metastasis or recurrence.

## 1. Introduction

Phyllodes tumours are rare fibroepithelial tumours that account for less than 1% of all breast tumours [1]. The term cystosarcoma phyllodes was coined by Johannes Müller, a misleading description as tumours are rarely cystic and the majority follow a benign clinical course. These tumours are predominantly seen in women aged 45–49 yrs [2, 3], rarely affecting adolescents and elderly [4, 5]. The term giant phyllodes is used when the tumour size exceeds 10 cm in maximum diameter [6].

While the surgical management of the phyllodes tumour has been addressed many times in the literature, few reports have specifically commented on the giant phyllodes tumour, an entity that presents the surgeon with several unique management problems. We hereby discuss and review the literature in managing giant malignant phyllodes.

## 2. Case Report

A 35-year-old lady presented with a large right breast mass since 6 months. There was no history of carcinoma breast in the family or in the past.

**Figure 1:**
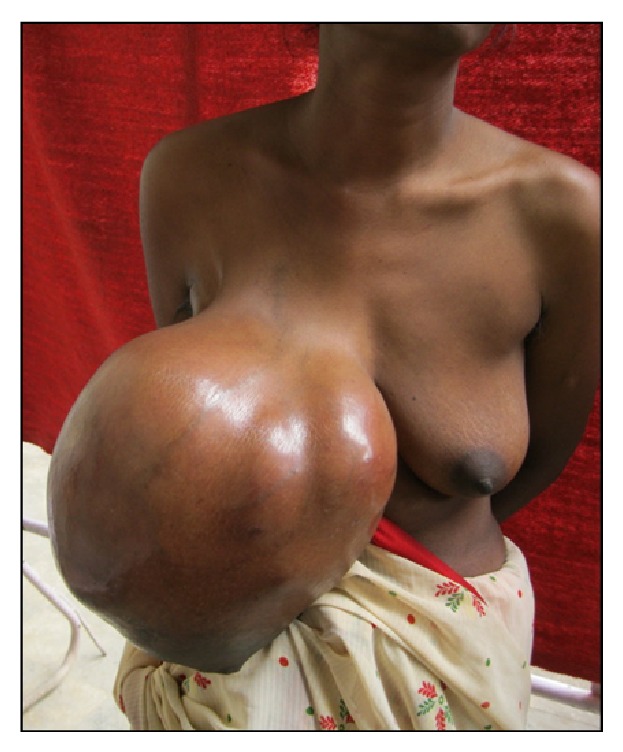
Anterior view of the tumour.

**Figure 2:**
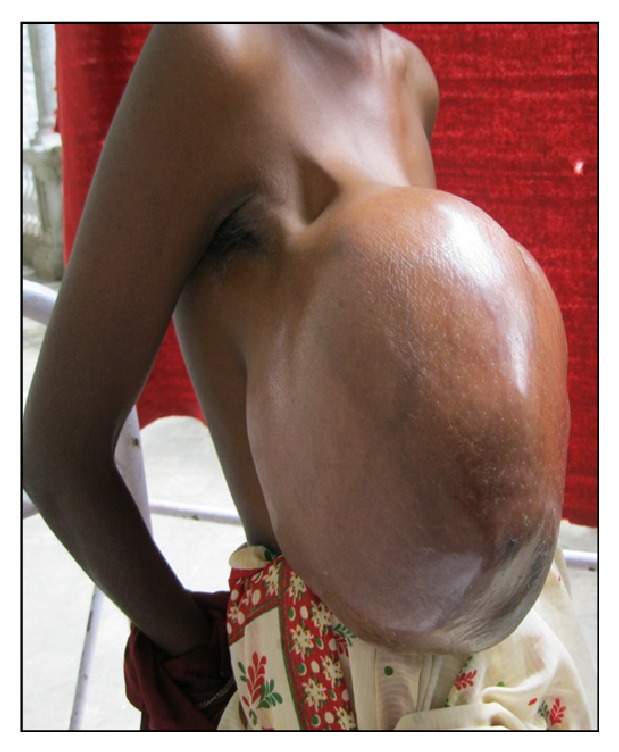
Lateral view of the tumour.

Examination revealed a large mass in the right breast measuring 36 × 32 cms with lobulated surface. Few dilated veins were noticed on the skin surface. On initial presentation there was no evidence of skin breakdown, but by the time of surgery patient had developed focal areas of skin necrosis. The nipple was pushed down inferomedially and was excoriated (Figures 1 and 2). Contralateral breast examination was normal, and there was no adenopathy on bilateral axillary examination.

Fine needle aspiration cytology revealed features suggestive of borderline phyllodes. Core biopsy examination showed biphasic neoplasm with malignant stromal component.

CT scan of brain and chest did not show any evidence of metastasis. Ultrasonography of abdomen was also found to be normal. Sonomammogram of right breast revealed fairly defined heterogenous lesion involving entire breast with minimal internal vascularity and multiple tubulocystic spaces within the lesion.

**Figure 3:**
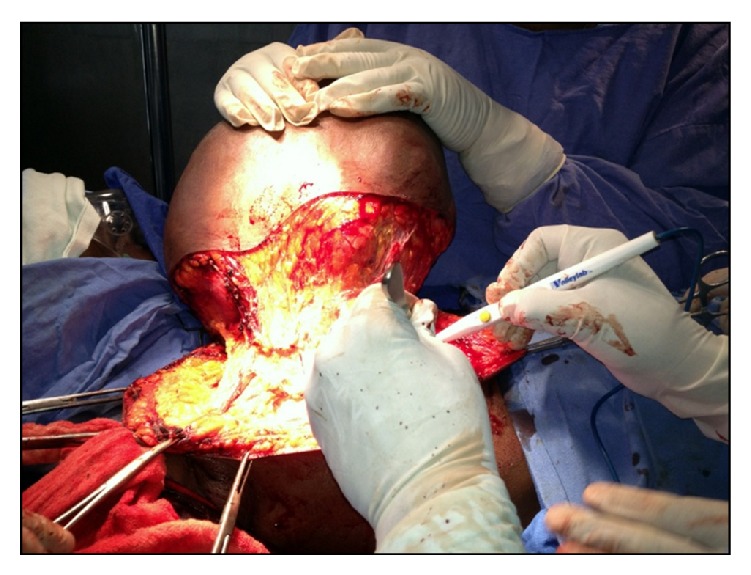
Intraoperative picture showing separation of the tumour from chest wall.

**Figure 4:**
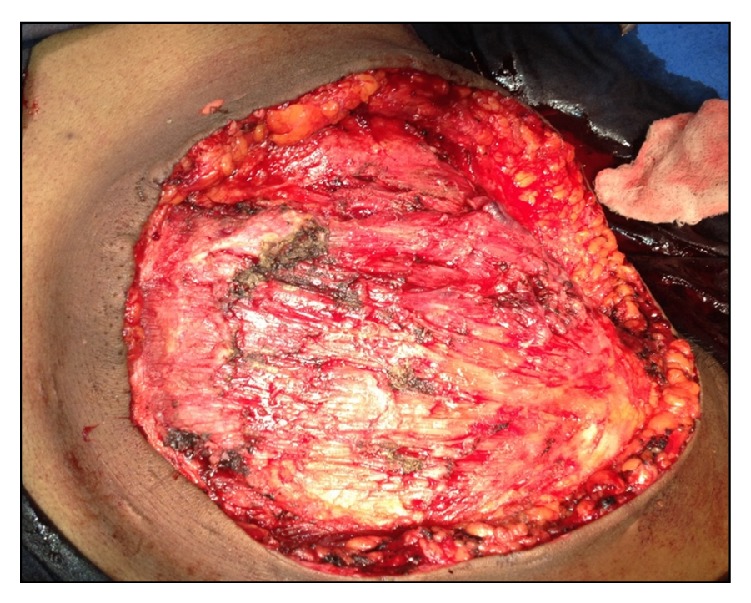
Chest wall after excision.

**Figure 5:**
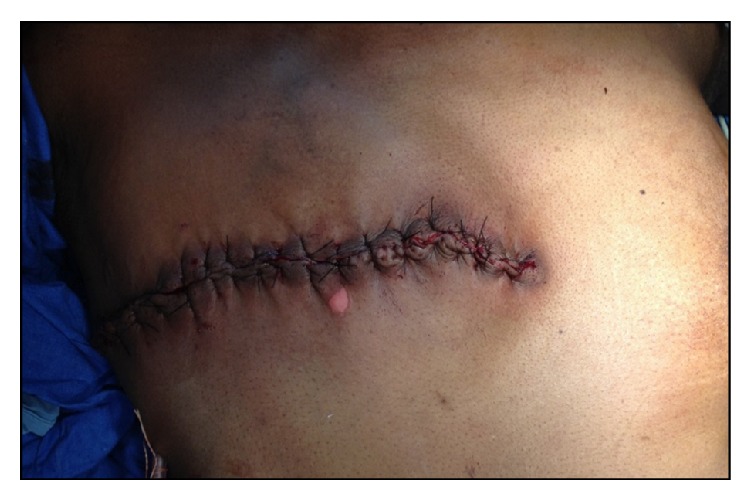
Postoperative picture.

**Figure 6:**
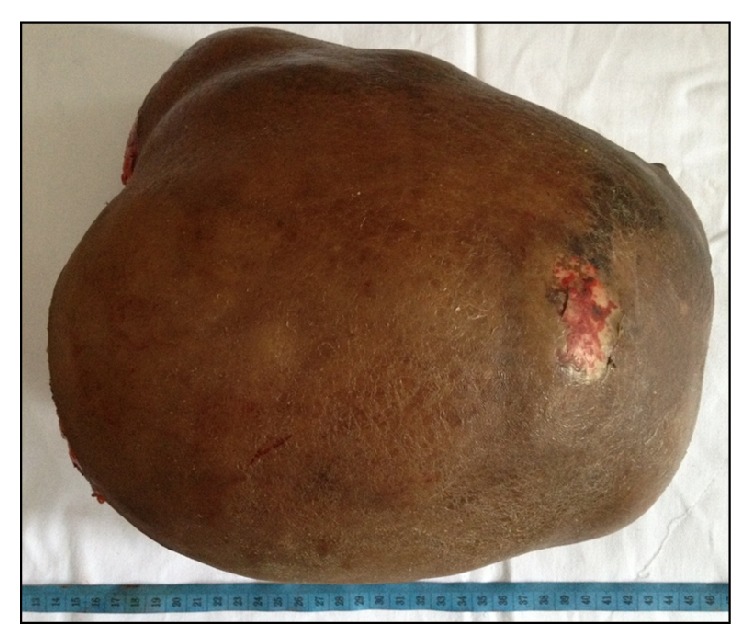
Gross specimen.

**Figure 7:**
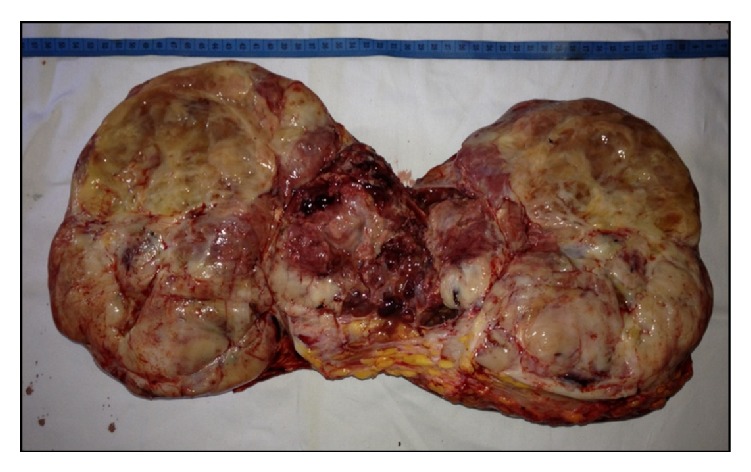
Cut surface of the specimen.

**Figure 8:**
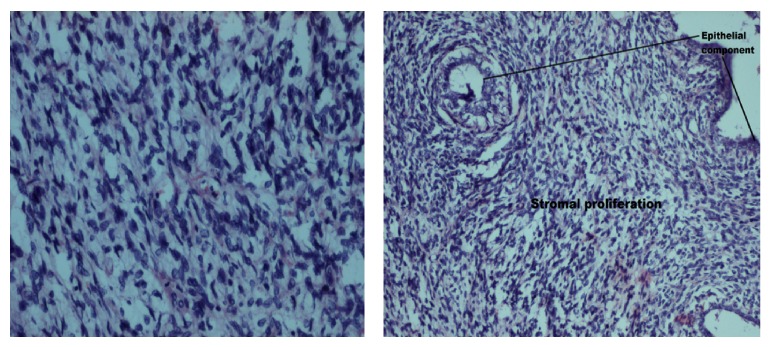
Histopathological picture showing high mitotic index with epithelial and high stromal proliferation.

Under cervical epidural anaesthesia, right simple mastectomy (Figures 3 and 4) was performed and incision closed primarily (Figure 5). Macroscopy revealed a lobulated tumour of size 33(L) × 32(B) × 22(D) cms, weighing 10 kgs (Figure 6). Cut section revealed multiple grey white lobulated fleshy areas with focal hemorrhagic and necrotic areas (Figure 7). Microscopic examination demonstrated high stromal cellularity and high mitotic rate > 10/10 hpf with moderate pleomorphism and extensive areas of tumour necrosis, confirming the diagnosis of malignant phyllodes tumour (Figure 8). All the resected margins were free of tumour. Based on histopathological examination a diagnosis of malignant phyllodes was made. Proliferation markers like Ki-67 and P53 were in the range of 12-13% and 5–7%, respectively.

Postoperatively the patient received doxorubicin and ifosfamide based chemotherapy 6 cycles with an interval of 28 days between each cycle, followed by radiotherapy of 50 Gy to the chest wall.

The patient is on one-year follow-up and shows no evidence of recurrence or metastasis.

## 3. Discussion

Phyllodes tumours are fibroepithelial neoplasms with epithelial and cellular stromal components, the latter of which represents the neoplastic process. The presence of an epithelial component differentiates the phyllodes tumour from other stromal sarcomas.

Classically patients present with a firm, mobile, well-defined round, macrolobulated, and painless mass. Large lesions may be associated with dilated veins visible over the skin, which may be stretched and attenuated. Nipple retraction [6, 9], skin ulceration invasion of the chest wall [6, 10, 11], and bloody nipple discharge [11] have also been reported but are rare.

Palpable axillary lymphadenopathy can be identified in up to 20% of patients at presentation, but metastatic involvement of axillary lymph nodes is rare, seen only in 2% [12], hence routine clearance of axillary lymphnodes is not recommended. These tumours spread hematogenously, with lung, pleura and bone being the most common sites of metastasis.

There are no pathognomonic mammographic or ultrasonographic signs in phyllodes tumour. In mammography, these lesions commonly present as voluminous isodense mass to breast parenchyma, usually greater than 5 cm, circumscribed, which may be associated with calcification. In a recent review examining the use of ultrasound in the diagnosis of phyllodes tumours, Chao et al. identified the following sonographic features that are characteristic of these tumours: well-circumscribed, lobulated masses, heterogeneous internal echo patterns, and a lack of micro calcifications [12].

Using fine needle aspiration (FNA) to diagnose a phyllodes tumour is associated with an excessive rate of false-negative results and an overall accuracy estimated at 63% [13]. Accuracy of FNA is often compromised by inadequate sampling because these tumours tend to have a very heterogeneous composition. Core biopsy, although subject to the same potential for sampling error, is associated with higher accuracy than FNA. In a recent article, Jacklin et al. [13] concluded that core biopsy is potentially the most useful method of preoperatively diagnosing phyllodes tumour. To help clinicians select patients for core needle biopsy, they have formulated a set of criteria that they refer to as the “Paddington Clinicopathologic Suspicion Score” which include the following.

*Clinical Findings*

- Sudden increase in size in a long-standing breast lesion.
- Apparent fibroadenoma >3 cm in diameter or in a patient >35 years.

*Imaging Findings*

- Rounded borders/lobulated appearance at mammography.
- Attenuation or cystic areas within a solid mass on ultrasonography.

*Fine Needle Aspiration Findings*

- Presence of hypercellular stromal fragments.
- Indeterminate features.

Any 2 features mandate core biopsy.

The intraoperative diagnosis of phyllodes tumour using frozen section is often inaccurate, similar to intraoperative frozen section diagnosis of other breast masses. Chen et al. [11] reported an accurate diagnosis using frozen section in only 41.6% of cases in their series of 172 patients with phyllodes tumour.

Microscopically, phyllodes tumours are characterised by epithelial lined cystic spaces with projection of hypercellular stroma into it. The presence of both epithelial and stromal elements is necessary to confirm the diagnosis. The stroma is the neoplastic component and determines the pathological behavior [14]. Only the stromal cells have the potential to metastasize [15].

Based on the histological characteristics of the tumour, including its margin (pushing or infiltrating), stromal cellularity (slight or severe), stromal overgrowth (absent, slight, or severe), tumour necrosis (present or absent), cellular atypia (absent, slight, or severe), and the number of mitoses per high power field, they can be classified into “benign,” “borderline,” and “malignant” categories. The widely accepted definitions as proposed by Azzopardi [7] and Salvadori et al. [3] are shown in Table 1. Other pathological classifications have defined similar categories but based on slightly different histopathological features [16, 17].

Wide local excision with margins greater than 1 cm is the preferred primary treatment [18]. However, an excision with the required margins is often impossible in giant phyllodes tumours such as in the case reported here. Mastectomy should be reserved for larger tumours [19, 20] and should be considered in recurrent tumours, especially of the malignant histotype [3, 21].

Local recurrence rates for phyllodes tumours are 15 to 20% and are correlated with positive excision margins, rather than with tumour grade or size [21]. Many histological prognostic factors have been evaluated. Different studies have regarded stromal overgrowth, tumour necrosis, infiltrating margins, mixed mesenchymal components, high mitotic rate, and stromal atypia as important, but in isolation each appears to have a low predictive value [1].

Local recurrence does not correlate with an increased risk for distant disease [11, 22] and does not seem to affect survival [3]. The literature is divided into the relationship between histologic grade and risk for local recurrence.

Some series suggest an increase in local recurrence among borderline and malignant lesions [3], but this has not been found in other large series [6, 11, 22, 23] (Table 2). In contrast, the development of metastatic disease has been shown to correlate with grade [6, 22, 24] and experts have estimated that 20% of patients with malignant tumours will develop metastatic disease [1]. Stromal overgrowth has been identified as an important independent histologic predictor of distant recurrence [24].

As studies of histological prognostic factors have been disappointing, recent interest has been shown in markers of tumour biology. p53 and Ki-67 expression correlate well with the morphologic gradings of PT.

There is no generalized accepted standard % to define “high expression.” Different authors applied different cut-off levels, from 5 to 34% for p53 and from 11.2 to 20% for Ki-67. However, all the authors concluded that expression of p53 and/or Ki-67 correlated with the morphologic grading [25].

The role of adjuvant treatments is unproven and must be considered on a case-by-case basis. In view of malignant phyllodes, this patient received both chemotherapy and radiotherapy.

Chaney et al. [24] proposed that adjuvant chemotherapy be considered for patients with histologic evidence of stromal overgrowth, particularly when the tumour size is greater than 5 cm, because these patients seem to have the greatest risk for developing distant disease.

The use of doxorubicin- and ifosfamide-based chemotherapy has shown some effectiveness [24] in cases of metastatic cystosarcoma phyllodes.

Pandey et al. [26] suggested that adjuvant radiotherapy also improved the disease-free survival. August and Kearney [27] recommended that adjuvant radiotherapy be considered for high-risk phyllodes tumours, including those >5 cm, with stromal overgrowth, with >10 mitoses/high power field, or with infiltrating margin.

The prognosis of phyllodes tumour is favourable, with local recurrence occurring in approximately 15% of patients overall and distant recurrence in approximately 5% to 10% overall [19].

The 5- and 10-year survival rates for malignant phyllodes tumour range from 54% to 82% and from 23% to 42%, respectively [27].

## 4. Conclusion

High-grade giant malignant phyllodes tumour is a very rare but aggressive breast malignancy. Stromal overgrowth carries a grave prognosis. Either wide local excision with adequate margins or mastectomy is an appropriate treatment for patients with malignant phyllodes tumour.

Adjuvant radiotherapy appears to improve disease-free survival and recurrence. Patients with high stromal overgrowth and >5 cm should be considered for systemic chemotherapy.

## Figures and Tables

**Table 1:** Criteria used to classify histological types as proposed by Azzopardi [7] and Salvadori et al. [8].

Category	Benign	Borderline	Malignant
Tumour margins	Pushing	←→	Infiltrative
Stroma cellularity	Low	Moderate	High
Mitotic rate (per 10 hpf)	<5	5–9	>10
Pleomorphism	Mild	Moderate	Severe

hpf: high power field.

**Table 2:** Prognostic factors implicated in the risk for distant and local recurrence in phyllodes tumour.

Prognostic factor	Implicated in the risk for local recurrence	Implicated in the risk for distant recurrence
Tumour size	No	Yes
Histologic grade	Unclear	Yes
Positive margin	Yes	—
Stromal overgrowth	—	Yes
Prior local recurrence	NA	No
